# Neuroprotective Effect of Ramipril Is Mediated by AT2 in a Mouse MODEL of Paclitaxel-Induced Peripheral Neuropathy

**DOI:** 10.3390/pharmaceutics14040848

**Published:** 2022-04-12

**Authors:** Hichem Bouchenaki, Amandine Bernard, Flavien Bessaguet, Simon Frachet, Laurence Richard, Franck Sturtz, Laurent Magy, Sylvie Bourthoumieu, Claire Demiot, Aurore Danigo

**Affiliations:** 1 UR 20218-NeurIT, Faculties of Medicine and Pharmacy, University of Limoges, 87025 Limoges, France; hichem.bouchenaki@gmail.com (H.B.); amandine.bernard@unilim.fr (A.B.); simon.frachet@unilim.fr (S.F.); laurence.richard@unilim.fr (L.R.); franck.sturtz@unilim.fr (F.S.); laurent.magy@unilim.fr (L.M.); sylvie.bouthoumieu@unilim.fr (S.B.); aurore.danigo@unilim.fr (A.D.); 2 INSERM 1083 CNRS UMR 6015 Mitovasc Laboratory, CarMe Team, University of Angers, 49045 Angers, France; flavien.bessaguet@univ-angers.fr; 3 Department of Neurology, Reference Center for Rare Peripheral Neuropathies, University Hospital of Limoges, 87000 Limoges, France; 4 Department of Biochemistry and Molecular Genetics, University Hospital of Limoges, 87000 Limoges, France; 5 Department of Cytogenetic, Medical Genetic and Reproduction Biology, University Hospital of Limoges, 87000 Limoges, France

**Keywords:** paclitaxel-induced peripheral neuropathy, angiotensin converting enzyme inhibitor, Ramipril, neuropathic pain, mouse model

## Abstract

Paclitaxel (PTX)-induced peripheral neuropathy (PIPN) induces numerous symptoms affecting patient quality of life, leading to decreased doses or even to cessation of anticancer therapy. Previous studies have reported that a widely used drug, ramipril, improves neuroprotection in several rodent models of peripheral neuropathy. The protective role of the angiotensin II type 2 receptor (AT2) in the central and peripheral nervous systems is well-established. Here, we evaluate the effects of ramipril in the prevention of PIPN and the involvement of AT2 in this effect. Paclitaxel was administered in wild type or AT2-deficient mice on alternate days for 8 days, at a cumulative dose of 8 mg/kg (2 mg/kg per injection). Ramipril, PD123319 (an AT2 antagonist), or a combination of both were administered one day before PTX administration, and daily for the next twenty days. PTX-administered mice developed mechanical allodynia and showed a loss of sensory nerve fibers. Ramipril prevented the functional and morphological alterations in PTX mice. The preventive effect of ramipril against tactile allodynia was completely absent in AT2-deficient mice and was counteracted by PD123319 administration in wild type mice. Our work highlights the potential of ramipril as a novel preventive treatment for PIPN, and points to the involvement of AT2 in the neuroprotective role of ramipril in PIPN.

## 1. Introduction

The occurrence of peripheral neuropathy with residual neuropathic pain is a major dose-limiting adverse effect of many commonly used chemotherapeutic agents and greatly affects patient quality of life. The only currently recognized prophylactic measure for chemotherapy-induced peripheral neuropathy (CIPN) is monitoring for pre-existing neuropathies and then the early detection of clinical symptoms of neuropathy in subjects undergoing neurotoxic chemotherapy treatment. The diagnosis of CIPN may justify either dose reduction or discontinuation, with an impact on cancer control and survival. Moreover, CIPN-associated persistent pain is related to the onset of several comorbidities including anxiety, depression, and insomnia, which have severe health consequences [1]. Thus, the prevention of CIPN and its improvement by an appropriate therapeutic approach is a key priority to avoid compromising anticancer treatments through dose reduction or cessation, as well as to improve patient quality of life. Paclitaxel (PTX) is the first member of the taxane family, one of the main neurotoxic classes of anticancer drugs. Paclitaxel is used to treat several types of cancer, including breast cancer, lung cancer and ovarian cancer. Among the taxane family, PTX shows the highest incidence of peripheral neuropathy [2], and induces the most severe CIPN symptoms [3]. Paclitaxel -induced peripheral neuropathy (PIPN) is responsible for predominantly painful sensory symptoms, including paresthesia or dysesthesia, mainly tactile allodynia, and shows a length-dependent pattern.

Considerable preclinical data has shown that RAS inhibitors, such as angiotensin-converting enzyme inhibitors (ACEi) and angiotensin receptor blockers (ARB), exert a neuroprotective effect in various murine models of peripheral neuropathy [4,5], such as traumatic [6,7,8], diabetic [9,10,11] and toxin-induced neuropathies [12]. A beneficial effect of ramipril, an ACEi, was demonstrated in animal models of chronic constriction injury, streptozotocin-induced diabetes, and oxaliplatin-induced acute pain syndrome [6,11,13]. Recently, Kim et al. reported that preventive or curative treatment with losartan, an ARB, delayed the onset of mechanical allodynia induced by PTX in rats [14]. We have previously demonstrated that the neuroprotective effect of candesartan, another ARB, in vincristine-induced peripheral neuropathy was mediated by the angiotensin II type 2 receptor (AT2) [15]. The AT2 receptor is one of the two main G-protein-coupled receptor subtypes of the RAS, along with AT1, and is expressed in numerous cells and tissues, notably in the sensory nervous system [16]. The protective role of AT2 in the central and peripheral nervous systems is well known. [4]. To our knowledge, the effect of preventive treatment with ramipril on PIPN has not been explored to date. In this study, we aimed to investigate the effect of ramipril in a mouse model of PIPN and the potential role of AT2 in this effect.

## 2. Materials and Methods

This study conformed with the guidelines for the ethical care of experimental animals of the European Community (2010/63/EU) and was approved by the French Ministry of Higher Education and Research (number 11280#2017091510483336). Experiments are reported in compliance with the ARRIVE guidelines [17]. All effort was made to limit suffering and minimize the number of animals used in the following experiments.

### 2.1. Treatments

Paclitaxel-induced peripheral neuropathy was induced by four intraperitoneal (i.p.) injections of PTX (1 injection every other day) (8 mg/kg/2 days; Hospira, France). An equivalent volume of PTX diluent was administered to control mice (physiological saline solution, i.p.). 

**Figure 1:**
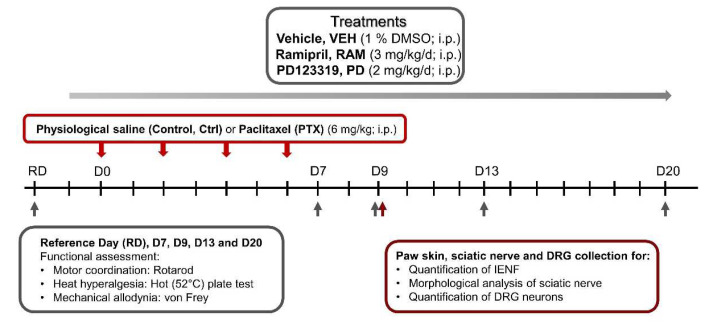
Study design. D: day, DMSO: dimethylsulfoxide, DRG: dorsal root ganglion, IENF: intraepidermal nerve fibers, i.p.: intraperitoneal.

Treatments with ramipril (3 mg/kg/day, i.p.; Sigma, Lezennes, France) and/or PD123319 (2 mg/kg/day, i.p.; Sigma, Lezennes, France) began one day ahead of the first PTX administration and were sustained daily for the next 20 days (Figure 1). This regimen has already been shown to have a neuroprotective effect in rodent models of traumatic peripheral neuropathy and oxaliplatin-induced acute pain syndrome [6,13]. Ramipril was diluted in a final solution of 1% dimethyl sulfoxide (1% DMSO) in physiological saline (0.9% NaCl). Vehicle mice received injections of an equivalent volume of 1% DMSO used for ramipril injections.

### 2.2. Animals

Male and female Swiss mice (6–7 weeks old, 25–30 g) from Janvier Labs (Le Genest-Saint-Isle, France) were housed in plastic cages and maintained on a 12-h light/dark cycle with food and water available ad libitum (BISCEm-animal care and facility center). 

Mice were randomized into four groups: vehicle (VEH), ramipril (RAM), PD123319 (PD) and ramipril combined with PD123319 (RAM+PD). Mice from each group were then randomly assigned to two subgroups: control (Ctrl) or paclitaxel (PTX), forming a total of eight groups of mice:

Ctrl-VEH
Ctrl-RAM
Ctrl-PD
Ctrl-RAM+PD
PTX-VEH
PTX-RAM
PTX-PD
PTX-RAM+PD

Mice were assigned to each group by using an online randomization tool (http://www.graphpad.com/quickcalcs/index.cfm accessed on 25 February 2019). All behavioral tests were assessed by the same investigator, blinded to the treatment (VEH, RAM, or PD) and the condition (PTX or Ctrl).

Functional tests were performed on the reference day (RD) to evaluate the baseline for each animal, on day 0 (D0) corresponding to the first PTX injection, then on D7, D9, D13 and D20 following the first PTX injection. Immunohistochemistry and morphological analyses were assessed on tissues removed three days following the final PTX injection, corresponding to the highest sensory impairment (Figure 1). 

To further elucidate the neuroprotective role of AT2, the same protocol was also performed using 5–6-week-old male and female C57BL6 AT2-deficient (AT2KO) mice. The AT2KO mice were randomly assigned to four groups: AT2KO-Ctrl-VEH, AT2KO-PTX-VEH, AT2KO-Ctrl-RAM and AT2KO-PTX-RAM. 

### 2.3. Behavioral Tests

#### 2.3.1. Motor Coordination

The rotarod test (Bioseb, Vitrolles, France) was performed to assess motor coordination. Mice were trained to walk against the motion of a rotating rod at a speed of 4 rpm three days prior to the start of the experiment (RD). On test days, mice were placed on the rotarod and subjected to a gradual 1 rpm/second acceleration for 30 s, following which the speed was maintained constant. The holding time (in seconds) was recorded. The cut-off time was established at 60 s. The mean holding time was calculated after each test session, which consisted of three trials separated by 5 min.

#### 2.3.2. Heat Hyperalgesia

The hot-plate test (Bioseb, Vitrolles, France) was used to evaluate thermal nociception [6]. Mice were placed on a 52 °C hotplate. The latency (s) time before the first withdrawal criterion including shaking, licking, or jumping was recorded. The cut-off was set at 30 s to avoid potential tissue damage. Mean latency was expressed as the threshold of an individual animal to thermal stimulation. Each test session consisted of three recordings separated by 5 min.

#### 2.3.3. Tactile Allodynia

Von Frey filaments (Bioseb, Vitrolles, France) were used to test tactile allodynia [9]. Mice were kept in a plastic cage with a wire mesh floor given accessibility to their paws to be examined. To avoid visual stimulation, the plastic cage was covered with an opaque cup. The mid-plantar left hind paw was examined. A modified version of the simplified up-down approach was used to determine the mechanical threshold [8]. A test round started with filament #6 (0.40 g) and progressed to higher or lower filaments values depending on the animal’s response. For each experimental condition, each animal went through three test rounds for each paw. The mechanical threshold was expressed as a percentage compared to the baseline measurement (%).

### 2.4. Immunohistochemistry of Footpad Skin and Dorsal Root Ganglia Neurons

To assess sensory innervation, animals (*n* = 6) were sedated by isoflurane then sacrificed by cervical dislocation. The footpads were then removed using punch biopsy (diameter 3 mm), fixed overnight in 4% paraformaldehyde solution (4% PFA), then cryoprotected (in 30% sucrose) and frozen at −20 °C. Sections of 20 µm were cut on a cryostat and incubated overnight at room temperature with the primary antibody to protein gene product 9.5 (PGP9.5, 1:50, Abcam, Paris, France). Sections were then incubated with a donkey anti-rabbit AF594-conjugated secondary antibody (diluted 1:500; Life Technologies, Saint-Aubin, France) for 2 h at room temperature. Epidermal nerve fibers were counted by blinded observers at 400× magnification (Eclipse 50i, Nikon Instruments, Nantes, France), according to established guidelines for human nerve counts [18]. Three slides per mouse were counted.

Four lumbar (L4 and L5) dorsal root ganglia (DRG) per mouse were collected to assess the DRG neuron density by counting neuron cellular bodies visualized by PGP9.5. Each DRG section (8 µm) was systematically photographed at 200× under a fluorescence microscope (Eclipse 50i; Nikon Instruments, Nantes, France). Immunoreactive DRG neurons were counted by blinded observers and the area containing neurons measured using NIS-Elements BR2.30 software (Nikon, Tokyo, Japan). The density of neurons positive for PGP9.5 is expressed as neurons/mm^2^. Three sections per DRG were counted.

### 2.5. Morphological Analysis of Sciatic Nerves

To assess the morphology and the quantification of myelinated and unmyelinated nerve fibers, sciatic nerves were dissected, fixed in 2.5% glutaraldehyde diluted in Sorensen buffer, then dehydrated, and embedded in Epon 812 resin (Euromedex, Souffelweyersheim, France). Semi-thin sections were stained with toluidine blue. Ultrathin sections were stained with uranyl acetate and lead citrate and observed by electron microscopy (JEM1400 Flash, Jeol, Peabody, MA, USA). A sufficient number of images to cover the entire section of the sciatic nerve was captured per animal (*n* = 3/group) at 3000× magnification and the number of myelinated fibers per mm^2^ counted to calculate the density.

### 2.6. Data Analysis

Data were analyzed using GraphPad Prism 8 software and expressed as the mean ± standard error of the mean (SEM). When statistical significance was identified by mixed-effects model statistical methods, individual comparisons were subsequently tested using Tukey’s multiple comparison tests for longitudinal follow-up of functional tests. For morphological analyses, a nonparametric Kruskal–Wallis test and Dunn’s multiple comparisons test were used to evaluate differences among multiple groups. Significance levels was represented as follows: * *p*-value < 0.05, ** *p* < 0.01, *** *p* < 0.001, **** *p* < 0.0001.

## 3. Results

### 3.1. Characterization of Paclitaxel-Induced Peripheral Neuropathy

#### 3.1.1. Paclitaxel Does Not Affect Motor Coordination and Heat Nociception

**Figure 2:**
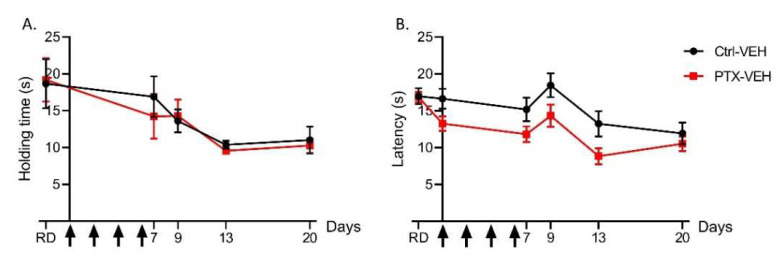
Motor coordination and heat nociception were not affected by PTX in wild type mice. (**A**) Motor coordination was evaluated by the rotarod test. The holding time reflects the capacity of mice to coordinate their movements in order to stay on the rod during acceleration (from 4 rpm to 40 rpm, 30 s). (**B**) Thermal nociception was evaluated by the hot plate test at 52 °C. *n* = 8 to 10. Black arrows indicate days on which PTX was administered. Ctrl: control, PTX: paclitaxel, RD: reference day, VEH: vehicle.

Motor coordination was evaluated using the rotarod test in the Ctrl-VEH and the PTX-VEH mice (Figure 2A). No significant difference in motor coordination between the PTX-VEH and the Ctrl-VEH mice was found at all test days. 

Heat nociception was evaluated in the PTX-VEH and the Ctrl-VEH mice using the hot plate test at 52 °C (Figure 2B). Though the data was suggestive of a light PTX-induced heat hyperalgesia, no significant difference in hot-plate withdrawal latencies was found between the PTX-VEH and the Ctrl-VEH mice. 

#### 3.1.2. Paclitaxel Induces Tactile Allodynia

**Figure 3:**
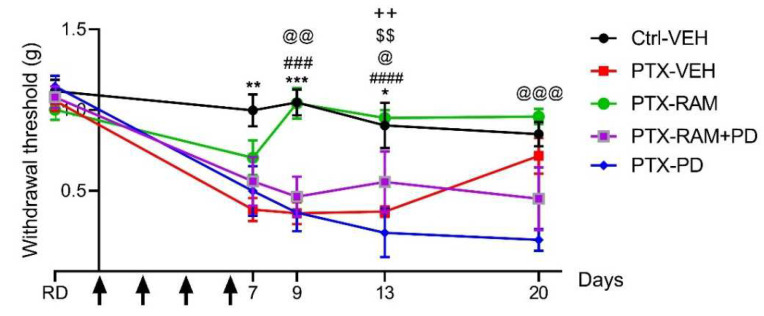
Ramipril prevents tactile allodynia induced by paclitaxel, this effect being counteracted by AT2 blockade, in wild type mice. Tactile sensitivity was evaluated by the von Frey filament test. *n* = 8 to 10. * *p* < 0.05, ** *p* < 0.01, *** *p* < 0.001 Ctrl-VEH vs. PTX-VEH. ^###^ *p* < 0.001, ^####^ *p* < 0.0001 PTX-VEH vs. PTX-RAM. ^@^ *p* < 0.05, ^@@^ *p* < 0.01, ^@@@^ *p* < 0.001 Ctrl-VEH vs. PTX-PD, ^$$^ *p* < Ctrl-VEH vs. PTX-RAM+PD. ^++^ *p* < 0.01 PTX-RAM vs. PTX-RAM+PD. Black arrows indicate days on which PTX was administered. AT2: angiotensin II type 2 receptor, Ctrl: control, PD: PD123319 (AT2 antagonist), PTX: paclitaxel, RAM: ramipril, RD: reference day, VEH: vehicle.

No difference in mechanical sensitivity was observed between any groups on the reference day. The PTX induced a significant tactile allodynia in wild type mice, manifested by lower mechanical thresholds from D7 to D13 (D7: *p* = 0.0018, D9: *p* = 0.0001, D13: *p* = 0.0378, PTX-VEH vs. Ctrl-VEH). The mechanical threshold returned to baseline 14 days after the last PTX injection (D20: *p* = 0.8471, PTX-VEH vs. Ctrl-VEH) (Figure 3). 

**Figure 4:**
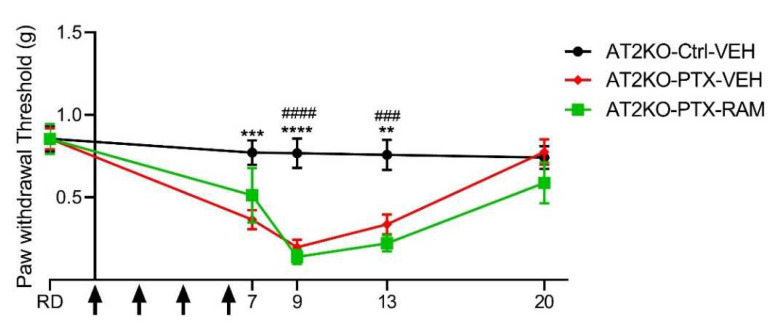
Ramipril did not show a neuroprotective effect in AT2-deficient (AT2KO) mice. Tactile sensitivity was evaluated by the von Frey filament test. *n* = 8 to 10. ** *p* < 0.01, *** *p* < 0.001, **** *p* < 0.0001 AT2KO-Ctrl-VEH vs. AT2KO-PTX-VEH. ^###^ *p* < 0.001, ^####^ *p* < 0.0001 AT2KO-PTX-VEH vs. AT2KO-PTX-RAM. Black arrows indicate days on which PTX was administered. Ctrl: control, PD: PD123319, PTX: paclitaxel, RAM: ramipril, RD: reference day, VEH: vehicle.

Tactile sensitivity was also evaluated in C57BL6 AT2KO mice (Figure 4). We observed that the baseline tactile sensitivity of the C57BL6 AT2KO mice was lower than in the Swiss wild type mice. Strain-dependent differences in sensitivity have already been observed in mice [19], thus this discrepancy could be due to the different genetic backgrounds of wild type and AT2-deficient mice, being Swiss and C57BL6 strains, respectively.

However, PTX also induced a significant tactile allodynia in the AT2KO mice from D7 to D13, as was found in the wild type mice (D7: *p* = 0.0007, D9: *p* < 0.0001, D13: *p* = 0.0028, AT2KO-PTX-VEH vs. AT2KO-Ctrl-VEH). Similarly, the mechanical threshold returned to baseline 14 days after the last PTX injection in AT2-KO mice (D20: *p* = 0.9469, AT2KO-PTX-VEH vs. AT2KO-Ctrl-VEH) (Figure 4).

#### 3.1.3. Paclitaxel Induces a Decrease in Sensory Neuron Terminals in the Epidermis, and a Decrease of Myelinated and Unmyelinated Nerve Fiber Densities in the Sciatic Nerve

**Figure 5:**
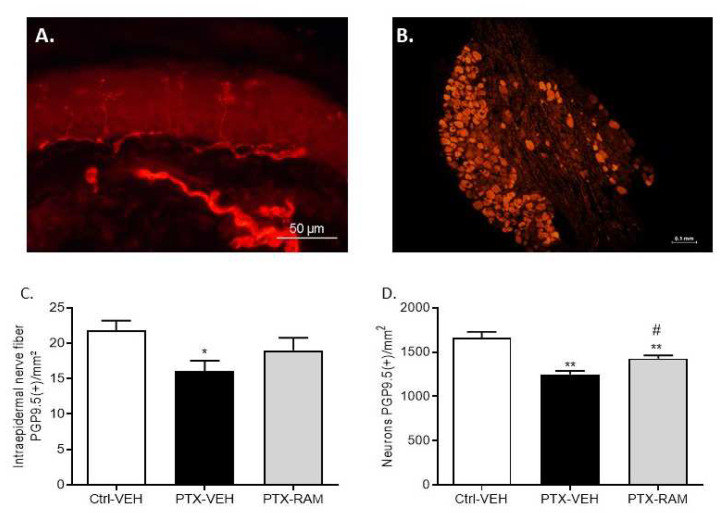
Ramipril alleviates sensory neuron loss induced by PTX administration in DRG of wild type mice. Immunohistochemistry for PGP9.5 was performed on paw skin (**A**) and DRG sections. (**B**). Intraepidermal nerve fiber density was assessed. Three sections of paw skin were examined per mouse. *n* = 6 mice (**C**). Quantification of DRG neuron density was evaluated. Three DRG sections and three DRG per mice were counted. *n* = 6 per group (**D**). * *p* < 0.05, ** *p* < 0.01, vs. Ctrl-VEH. ^#^ *p* < 0.05, PTX-VEH vs. PTX-RAM. Ctrl: control, PTX: paclitaxel, RAM: ramipril, VEH: vehicle.

Paw skin and DRG sections were stained with PGP9.5, a pan-neuronal marker, for evaluating densities of IENF and of DRG neurons, respectively (Figure 5A,B). A significant difference in IENF density, corresponding to sensory nerve endings, was found between the Ctrl-VEH and the PTX-VEH groups (*p* = 0.0210) (Figure 5C). Similarly, the density of DRG neurons, corresponding to the cell body of sensory neurons, was significantly reduced by PTX administration (*p* = 0.0002, PTX-VEH vs. Ctrl-VEH) (Figure 5D).

**Figure 6:**
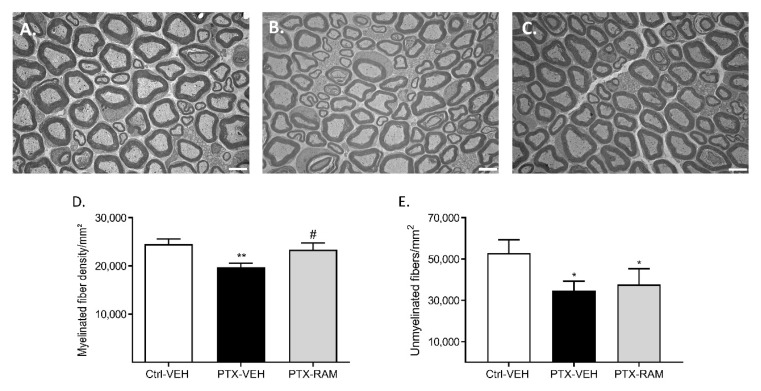
Ramipril alleviates PTX-induced loss of myelinated fibers in the sciatic nerve of wild type mice. (**A**–**C**) are representative images of sciatic nerve sections visualized by electron microscopy from the Ctrl-VEH, PTX-VEH and the PTX-RAM groups, respectively. (**D**) Quantification of myelinated fiber density. (**E**) Quantification of unmyelinated fiber density. *n* = 6 per group. * *p* < 0.05, ** *p* < 0.01, vs. Ctrl-VEH. ^#^ *p* < 0.05, PTX-VEH vs. PTX-RAM. Ctrl: control, PTX: paclitaxel, RAM: ramipril, VEH: vehicle.

Sciatic nerves were removed and processed for electron microscopy. Transverse sections were used to quantify densities of myelinated and unmyelinated fibers (Figure 6). Paclitaxel led to a significant decrease in myelinated nerve fiber density in the sciatic nerve (*p* = 0.0016, PTX-VEH vs. Ctrl-VEH) (Figure 6D). The density of unmyelinated nerve fibers was also significantly lower in the PTX-VEH group compared to the Ctrl-VEH group (*p* = 0.0106) (Figure 6E).

### 3.2. Effect of Ramipril and Involvement of AT2 on PIPN

#### 3.2.1. Ramipril Accelerates Recovery of Normal Mechanical Sensitivity in Wild Type Mice and this Effect Was Counteracted by Blockade of AT2

Treatment with ramipril, PD123319 or with a combination of both did not affect the mechanical sensitivity in Ctrl mice (data not shown). Mice receiving PTX and ramipril did not develop mechanical allodynia. The sensitivity of the PTX-RAM mice was similar to the Ctrl-VEH mice and significantly lower than the PTX-VEH mice from D9 to D13 (D9: *p* = 0.0002, D13: *p* < 0.0001, PTX-VEH vs. PTX-RAM) (Figure 3). The beneficial effect of ramipril on PTX-induced mechanical allodynia was lost when combined with PD123319. Treatment of PTX-administered mice with a combination of ramipril and PD123319 did not prevent the onset of mechanical allodynia induced by PTX (D13: *p* = 0.0062, Ctrl-VEH vs. PTX-RAM+PD, *p* = 0.0054, PTX-RAM vs. PTX-RAM+PD). Mice in the PTX-RAM+PD group developed mechanical allodynia, which was similar to mice in the PTX-VEH group (D7: *p* = 0.7230, D9: *p* = 0.8819, D13: *p* = 0.7753, D20: *p* = 0.6468). The blockade of AT2 by PD123319 did not influence the onset of PTX-induced mechanical allodynia (D9: *p* = 0.0019, D13: *p* = 0.0382, Ctrl-VEH vs. PTX-PD). Moreover, mechanical allodynia induced by PTX appeared to be maintained by PD123319 (D20: *p* = 0.0127, PTX-VEH vs. PTX-PD). In fact, the mechanical sensitivity of mice from the PTX-RAM+PD and PTX-PD groups did not return to baseline at D20 as was seen in the PTX-VEH group (D20: *p* = 0.0001, Ctrl-VEH vs. PTX-PD).

#### 3.2.2. Ramipril Accelerates Recovery of Normal Mechanical Sensitivity in Wild Type but Not in AT2KO Mice

Evaluation of mechanical sensitivity was also performed in AT2-deficient mice to confirm its involvement in the preventive effect of ramipril on the onset of PTX-induced mechanical allodynia (Figure 4). The AT2-deficient mice treated with PTX in combination with Ramipril developed a significant mechanical allodynia from D9 to D13, similar to that observed in AT2-deficient mice treated only with PTX (D9: *p* < 0.0001, D13: *p* = 0.0002, AT2KO-Ctrl-VEH vs. AT2KO-PTX-RAM). Thus, the preventive effect of ramipril was completely lost in AT2-deficient mice.

#### 3.2.3. Ramipril Ameliorates Morphological Alterations Induced by PTX

Ramipril did not affect IENF and DRG neuron densities in the control groups (data not shown). The decrease in IENF density caused by PTX was ameliorated by ramipril. Indeed, though no significant difference was observed between the PTX-VEH and the PTX-RAM groups (*p* = 0.1201), it should be noted that there was also no longer a difference between the Ctrl-VEH and the PTX-RAM groups in IENF density (*p* = 0.1838) (Figure 5A). Ramipril did not completely prevent but did significantly improve the decrease in DRG neuron density induced by PTX (*p* = 0.004, Ctrl-VEH vs. PTX-RAM; *p* = 0.0159, PTX-VEH vs. PTX-RAM) (Figure 5B).

Ramipril had no effect on the morphology of myelinated or unmyelinated nerve fibers in the sciatic nerves of the control groups (data not shown). No notable changes in unmyelinated fiber morphology were observed in the PTX-VEH mice compared to the Ctrl-VEH mice. Ramipril significantly prevented the decrease of myelinated fiber density that is induced by PTX (*p* = 0.022, PTX-RAM vs. PTX-VEH) (Figure 6A). In contrast, ramipril treatment had no effect on the loss of unmyelinated fibers induced by PTX (*p* = 0.0472, Ctrl-VEH vs. PTX-RAM; *p* = 0.4415, PTX-VEH vs. PTX-RAM) (Figure 6B).

## 4. Discussion

The main findings of this study are that: (1) the mouse model of PIPN which we used was characterized by mechanical allodynia, associated with a decrease in the IENF density, and degeneration of the DRG neurons and myelinated nerve fibers in the sciatic nerve, (2) ramipril prevented the onset of PTX-induced mechanical allodynia, and partially ameliorated the alterations in morphology, and (3) the neuroprotective effect of ramipril on PTX-induced allodynia was mediated by AT2.

The experiments conducted on PTX- and VEH-treated mice showed that administration of PTX did not alter motor coordination, as assessed by the rotarod test. This suggests that PTX does not affect the peripheral nerves involved in motor function, i.e., large Aα-type myelinated fibers. This observation is consistent with the fact that large myelinated sensory nerve fibers have also been reported to be the most susceptible to PIPN in human [20]. Previous reports have highlighted alterations of thermal sensitivity in PIPN rodent models including cold allodynia [21,22], heat hyperalgesia [23] and heat hypoalgesia [24,25,26]. In our experimental conditions, we did not notice any alterations in heat nociception induced by PTX administration. Differences in doses, formulations and injection schedule of chemotherapy drugs, and the use of different mouse strains as well as the varying endpoints measured by behavioral tests, can lead to considerable differences in outcomes between two similar models [19,27]. In this regard, and to improve translational research in the case of CIPN, we previously discussed the urgent need to standardize the methods used to evaluate pain in animal models [28]. Lastly, PTX administration did lead to the onset of mechanical allodynia as evidenced by results of the von Frey test. 

In the various murine models of PIPN that have been previously developed, mechanical hypersensitivities are the most common observed and predominant functional alterations [19,21,22,24,29,30]. Accumulation of PTX in DRG neurons has been demonstrated in several rodent models of PIPN, causing a distal-to-proximal degeneration of peripheral sensory nerve fibers, preferentially in myelinated nerve fibers, but also, and to a lesser extent, causing degeneration of the central branches of DRG neurons [31,32]. Mechanical allodynia is a complex process involving many players in the central and peripheral nervous systems. Paclitaxel -induced mechanical allodynia may be mediated by components of the PNS, and more particularly by an alteration in myelinated Aβ fibers (mechano-nociceptors) and unmyelinated C fibers (polymodal nociceptors), both of which have been shown to be involved in the development of mechanical allodynia in previous studies [33,34]. Additionally, central injuries at the spinal cord level may be involved [35]. In fact, as is the case with oxaliplatin, patients receiving PTX commonly develop painful symptoms a few hours after the first PTX administration, without overt evidence of nerve degeneration, and these early defects have been designated PTX-induced acute pain syndrome [36,37]. In our study, morphological analyses showed that PTX treatment led to a decrease in IENF density, a loss of sensory DRG neurons, and loss of myelinated and unmyelinated nerve fibers in the sciatic nerves, corresponding to the length-dependent sensory axonopathy observed in patients experiencing PIPN [38,39]. Thus, the Swiss mouse model used in our study, characterized by sensory deficit and nerve degeneration reproduced the clinical features of chronic PIPN. 

Daily administration of ramipril prevented the development of PTX-induced mechanical allodynia and morphological changes in nerves. Blockade of the renin-angiotensin system with ACE inhibitors has already been shown to have analgesic effects in humans and in several models of neuropathic pain [5,40] induced by chronic constriction injury [6,8], insulin resistance [41], or streptozotocin-diabetes [9]. However, our results are in opposition to those of a previous study which reported that enalapril, an ACE inhibitor, potentiated mechanical allodynia in a mouse model of PTX-induced pain syndrome [42]. In this study, very low doses of PTX (0.001 to 0.004 mg/kg, i.p.) were administered leading to the onset of mechanical allodynia without overt evidence of neurodegeneration. We could suggest that ACE inhibitors may be proalgic in the first hours following a single administration, and then become neuroprotective and analgesics in the long run, in the case of neuropathic pain associated with structural changes to nerves. Moreover, the authors demonstrated that potentiation of allodynia by enalapril was mediated by B1 and B2 kinin receptors [42]. Another study showed that treatment with ramipril inhibited tactile and cold allodynia, and reduced the expression of the pro-nociceptive kinin B1 receptor in a rat model of sensory neuropathy induced by glucose-feeding [41]. In this latter model, as well as in ours, the beneficial effect of ramipril could be associated with its anti-inflammatory and antioxidant effects, independently of the kinin system. Indeed, both processes, inflammation and oxidative stress, participate in the development of PIPN [43]. Ramipril has previously been shown to have beneficial effects in a model of neuropathic pain induced by CCI, and these effects were associated with anti-inflammatory and antioxidant actions, following a decrease in Ang II levels [6]. Therefore, it cannot be excluded that anti-inflammatory and antioxidant effects of ramipril were attributable to its role in RAS modulation. However, further investigations are now needed to explore the involvement of the kinin system in the effects of ramipril, under the same experimental conditions as were used here.

We have previously shown that direct or indirect stimulation of AT2 prevents mechanical allodynia induced by VCR [15]. Moreover, the neuroprotective role of AT2 in the central and peripheral nervous system has also been the subject of numerous studies and has been reviewed elsewhere [4,44]. In view of this, we tested the hypothesis that the protective effect of ramipril in PTX-induced neuropathy is mediated by AT2. Blockade of AT2 by PD123319 was found to counteract the beneficial effect of ramipril. PD123319 is an AT2 antagonist, 10,000-fold more selective for AT2 than for AT1, however, PD123319 is also a putative ligand for Mas-related receptor (Mgrd) [45]. The use of AT2KO mice showed a complete loss of the preventive effect of ramipril on PTX-induced mechanical allodynia, and so confirm the involvement of AT2 in the ramipril effect. Thus, using both pharmacological and gene deficiency approaches, our results strongly suggest that the beneficial effect of ramipril on PTX-induced mechanical allodynia was mediated by stimulation of AT2. One hypothesis is that the blockade of ACE by ramipril could favor the ACE2 pathway which cleaves Ang I to produce Ang (1–9), and which interacts with AT2. The Ang (1–9)/AT2 axis has mostly been investigated for its cardioprotective role, which has been previously reviewed elsewhere [46]. In addition, the anti-inflammatory effects of AT2 stimulation have been documented in many tissues and may in our case participate in the antiallodynic and neuroprotective effects of ramipril [47]. Stimulation of AT2 also reduces reactive oxygen superoxide (ROS) production in M1 activated macrophages which are classically defined as proinflammatory [48]. There is evidence that PTX treatment causes macrophage accumulation in the DRG and sciatic nerves in rodents, thus contributing to the accumulation of ROS and development of CIPN [49,50].

Several studies have reported an analgesic effect following AT2 blockade [51,52,53]. Moreover, one study has reported that blockade of AT2 by PD123319 (at a single dose of 20 mg/kg) prevented mechanical and cold allodynia in a mouse model of PTX-induced acute pain syndrome [54]. In our experimental conditions, i.e., in a mouse model of chronic PIPN, the blockade of AT2 maintained mechanical allodynia as sensitivity of the mice treated with PD123319 did not return to baseline as was the case in untreated PTX mice. This discrepancy could be explained by the difference of doses and schedule of administration. Indeed, AT2 agonism has already showed neuroprotective/neuroregenerative effects in other models [4]. In this way, the blockade of AT2 by daily administration of PD123319 on a long period (20 days) could inhibit some neuroregenerative processes and thus affect the recovery of normal tactile sensitivity.

To conclude, we have shown that ramipril administration alleviates functional and morphological alterations induced by PTX in our mouse model of sensory neuropathy. Moreover, we propose an alternative and novel mechanism for ramipril-mediated neuroprotection that is mediated by AT2 activation. We suggest that stimulation of AT2 represents a possible therapeutic strategy in the case of long-term effects associated with CIPN. As ramipril is a relatively inexpensive, safe and well-tolerated drug [55,56], our results encourage future clinical evaluation of the preventive therapeutic potential of ramipril in patients exposed to PTX-induced sensory neuropathy.

## Data Availability

Not applicable.
